# Control of (Multi)Drug Resistance and Tuberculosis Incidence over 23 Years in the Context of a Well-Supported Tuberculosis Programme in Rural Malawi

**DOI:** 10.1371/journal.pone.0058192

**Published:** 2013-03-06

**Authors:** Sebastian M. Mboma, Rein M. G. J. Houben, Judith R. Glynn, Lifted Sichali, Francis Drobniewski, James Mpunga, Paul E. M. Fine, Neil French, Amelia C. Crampin

**Affiliations:** 1 Karonga Prevention Study, Chilumba, Malawi; 2 London School of Hygiene and Tropical Medicine, London, United Kingdom; 3 National TB Control Programme, Lilongwe, Malawi; 4 University of Liverpool, Liverpool, United Kingdom; 5 Health Protection Agency National Mycobacterium Reference Laboratory, London, United Kingdom; University of California, San Francisco, United States of America

## Abstract

**Background:**

The rise in tuberculosis (TB) incidence following generalized HIV epidemics can overwhelm TB control programmes in resource-limited settings, sometimes accompanied by rising rates of drug resistance. This has led to claims that DOTS-based TB control has failed in such settings. However, few studies have described the effect of a sustained and well-supported DOTS programme on TB incidence and drug resistance over a long period. We present long-term trends in incidence and drug resistance in rural Malawi.

**Methods:**

Karonga District in northern Malawi has an adult HIV prevalence of ∼10%. A research group, the Karonga Prevention Study, collaborates with the National Tuberculosis Programme to support core TB control activities. Bacteriological, demographic and clinical (including HIV status) information from all patients starting TB treatment in the District have been recorded since 1988. During that period isolates from each culture-positive TB patient were exported for drug sensitivity testing. Antiretroviral therapy (ART) has been widely available since 2005.

**Results:**

Incidence of new smear-positive adult TB peaked at 124/100,000/year in the mid-90s, but has since fallen to 87/100,000/year. Drug sensitivity information was available for 95% (3132/3307) of all culture-positive cases. Initial resistance to isoniazid was around 6% with no evidence of an increase. Fewer than 1% of episodes involved a multi-drug resistant strain.

**Discussion:**

In this setting with a generalised HIV epidemic and medium TB burden, a well-supported DOTS programme enhanced by routine culture and drug sensitivity testing may well have reduced TB incidence and maintained drug resistance at low levels.

## Introduction

The HIV epidemic caused a dramatic increase in tuberculosis (TB) incidence in sub-Saharan countries. [1] In South Africa the HIV-driven rise in TB incidence was accompanied by escalating rates of TB drug resistance. [2] However, few data are available from other sub Saharan countries [3], [4], especially on trends in drug resistance. [5]–[7].

The current debate around TB control is focusing on the rapid scale-up of improved, but costly, new diagnostics as a measure essential to TB control in HIV endemic settings. [8]–[12] Unfortunately, infrastructural and financial restrictions make the implementation of currently available tests, such as GeneXpert^©^, highly unlikely at the peripheral level in low income settings. [13] TB control programmes in these areas, where the majority of people at risk of TB live, may require an alternative approach, which could include strengthening existing DOTS-based systems.

WHO’s 2010 global report showed signs that overall TB incidence rates are beginning to fall in the African region outside South Africa [14], and that, despite their limited resources, the standard DOTS, smear-microscopy based programmes in the area are starting to control TB. Cross sectional surveys also suggest that drug resistance levels are relatively low in much of Africa. [3]–[6].

We report long-term trends in TB incidence and drug resistance in the context of a well-supported TB programme in Karonga District, a rural district in northern Malawi [15], where an effective ART programme is in place. [16] Previous reports from this area showed a HIV-induced rise of TB incidence during the 1990s, [17] and stable drug resistance over that period. [18]–[20] We now present TB incidence and drug resistance trends during a further decade of TB control.

## Methods

### Ethics Statement

Informed consent was sought from all participants, including separate counselling and consent for HIV testing. In the earlier years of the study, data were collected in the context of providing a clinical and audit service for the TB control programme and consent was verbal, in line with norms of operational research at the time. A record of whether individual TB patients were willing or unwilling to participate in data collection or to accept HIV testing and counselling, was held in hand-written TB patient registers owned by the National TB programme. Those patients who also participated in trials conducted during that period, were asked for written consent by the Karonga Prevention Study (KPS). Since 2001, all TB patients have been asked for written consent for routine procedures, if undertaken by KPS rather than government staff. All written consent forms are stored on site at the KPS headquarters in Chilumba, Malawi. Ethical approval for the study, including consent procedures at each time point, was received from the London School of Hygiene & Tropical Medicine and the Malawian National Health and Sciences Research Committee (current approval numbers LSHTM #5067 and NHSRC #424).

### Study Setting

Karonga District covers 3355 km2 and its current total population is estimated around 300,000. [21] Adult HIV prevalence peaked at 12% in the mid-1990s, and is currently stable around 10% ([22] & unpublished data). The first ART clinic opened in 2005 [23] and as of June 2011, over 5000 individuals were receiving ART from 6 clinics. In collaboration with the National Tuberculosis Programme (NTP), a research group, the Karonga Prevention Study (KPS) has been supporting core TB programme activities and conducting TB epidemiological studies of TB in Karonga District since 1986. [15].

### Patients and Case Finding

Case finding procedures have changed over time for study purposes, and are summarized in table 1. In 1986–89 a total population survey was conducted as the recruitment phase of a vaccine trial of repeat BCG and killed *M.leprae*. [24], [25] Everyone seen was asked about cough and examined for lymphadenopathy.

**Table 1:** Tuberculosis and other activities in Karonga District - 1986–2012.

Case finding																													
Population survey		###############																											
Passive case finding in clinics			##########			##################					##################						##################					##################					############		
Short surveys of intensified case finding^a^																									#######				
Annual cough screening on patients with previous TB																								##########			############		
Cough screening in demographic surveillance area																			##########			##################					############		
Diagnosis																													
Ziehl Neelsen smear microscopy		###############				##################					##################						##################					##################					############		
Auramine smear microscopy									#######		##################						##################					##################					############		
Culture confirmation^b^		###############				##################					##################						##################					##################					############		
Drug sensitivity testing^b^		###############				##################					##################						##################					##################					############		
Treatment																													
Treatment delivered by KPS staff				#######		##################					##################						##################												
Other activities																													
HIV testing on TB cases			##########			##################					##################						##################					##################					############		
ART available^c^																						##################					############		
Year	86				90					95						00					05					10			12

a 1997–1999: survey for lymph node TB in district hospital. 2008–2009: general TB screening of patients on general ward of district hospital.

b Species confirmation and dug sensitivity tested in UK laboratories. Done for study purposes, seldom used in diagnostic decision (i.e. start tuberculosis treatment or not) due to delay in sending sample and receiving result.

c mostly based on clinical staging. When CD4 was available, ART eligibility threshold was raised from <200 to <250 in 2007, and to <350 in 2011.

From the end of the survey to 2007 KPS field staff were based in all health centres, where one of their functions was to take sputum samples from individuals presenting with chronic cough or lymphadenopathy. Three sputa were collected following a spot-morning-spot schedule. [26] If the individual did not present at the clinic the next day, the field worker would collect sputa at the household. Since 2007, field workers have only been present one morning a week at the small health centres (their work is now covered by local health staff). KPS still has a continuous presence at the three main clinics.

From 1997–99 a study of lymph node TB increased the number of biopsies and fine needle aspirates taken from suspected cases. [27] An immunological study, started in 2008, recruited patients from the general ward in the district hospital. These patients were screened for TB as part of the protocol, regardless of symptoms, thus potentially intensifying case finding on the ward.

Participants in other community-based KPS studies in the District are also asked about persistent cough. [15] This screening has a low yield, suggesting that case detection through the routine measures is good.

### Laboratory Diagnosis

Samples are transported to the KPS laboratory in the south of the district. All samples are investigated for mycobacteria using smear microscopy, initially with Ziehl-Neelsen staining only, but since 1996 all smears have been screened using auramine-stained fluorescence microscopy, with Ziehl-Neelsen confirmation of positives.

Results from the KPS laboratory are reported back to the field worker who took the samples and to the NTP. When positive, the result is delivered to the household if the individual does not return to the health centre to collect it, and the patient is referred for TB treatment. If a person does not present to the clinic, they are again visited at home to encourage them to come for treatment.

Specimens are cultured on Lowenstein-Jensen media for the isolation of Mycobacterium species. Patients are started on treatment if a culture growth suggests *M.tuberculosis.*

Species confirmation and drug sensitivity testing are done at the HPA National Mycobacterium Reference Laboratory in the United Kingdom. Samples are sent in batches to minimize international transport costs. Final results, therefore, typically arrive at least 12 weeks after sputum is first taken and submitted, but can sometimes take much longer. Drug resistance is tested using the ratio method. [28] Until 2001, all first line drugs were tested and if resistance was found, further sensitivity testing for second line drugs was carried out. From 2001 onwards, samples were initially tested for resistance to isoniazid and rifampicin only, with further testing if resistance was seen to either of these drugs. All isolates from culture-positive patients from 1996 to 2008 have undergone molecular typing using IS*6110* RFLP. [29]–[30].

### Treatment and Follow-up

Until 2004, KPS acted on behalf of the NTP in collaboration with the District TB officer to register and distribute treatment to patients. Since 2004, this task has been managed by the NTP. Since 1988 all TB patients have been offered HIV testing by KPS staff and since 2005 they have been referred for ART screening when HIV-positive. Eligibility for ART relied mainly on clinical staging as WHO stage 3 or 4; CD4 was not widely available. For those with access to CD4 testing, the level at which they were eligible for ART increased from 200 to 250 in 2007, and to 350 in 2011.

TB treatment regimens have followed NTP guidelines throughout the study period, and currently the standard DOTS treatment is given to all patients (see table 2). [31] Rifampicin has been part of the intensive treatment phase since 1984 and has been given throughout treatment since 2007. [31].

**Table 2:** TB treatment regimen in Malawi during the study period.

Patient Category	Period and Regimen			
	1984–1995	1996–2000	2001–2006	2007 to present
New TB patients				
Adult* smear-positive pulmonary including military TB; Extra-pulmonary TB of the spine and pericardium	2SRHZ/6HT_3_	2SRHZ/6HE	0.5RHZE/1.5R_3_H_3_Z_3_E_3_/6EH	2RHZE/4RH
Adult smear-negative TB and other extra-pulmonary TB not included above	1SEH/11HT_3_	1SEH/11HE	0.5RHZE/1.5R_3_H_3_Z_3_E_3_/6EH	2RHZE/4RH
Children*	2SRHZ/6HT_3_	5R_3_H_3_Z_3_/6EH*	0.5(RH)HZE/1.5(RH)_3_H_3_Z_3_E_3_/6EH*	2RHZ/4RH*
TB meningitis	2SRHZ/7RH	2SRHZ/7RH	2SRHZ/7RH	2SRHZ/7RH
Previously treated for TB				
Recurrent TB, TB treatment failures and Defaulters	2SRHZE/1RHZE/5HT_3_	2SRHZE/1HRZE/5R_3_H_3_Z_3_E_3_	2SRHZE/1RHZE/5R_3_H_3_Z_3_E_3_	2SRHZE/1RHZE/5RHE

* Adult = 15 years or older, child = less than 15 years old.

S = streptomycin; R = rifampcin; H = isoniazid; Z = pyrazinamide; T = thiacetazone; E = ethambutol. Large numbers indicate duration in months. All drugs taken daily, unless subscript indicates alternative number of doses a week.

Second line anti-TB drugs were not available in Malawi until 2007. [31] Initially, the NTP recommended use of isoniazid and ethambutol for patients with multidrug resistant (MDR) TB. Since 2007, MDR-TB has been treated with six months of pyrazinamide, cycloserine, ethionamide, ofloxacin and an injection of capreomycin, followed by eighteen months of the same drugs without capreomycin. These drugs have to be requested by the District TB coordinator directly from central medical stores following a laboratory confirmation of MDR TB. In this setting patients are often close to completion of first line treatment by the time MDR TB is confirmed due to the time taken for culture and the batching of cultures for international transportation.

The procurement, distribution and dispensing of anti-tuberculosis drugs in Malawi are strictly regulated, with sole authority given to the NTP. Private health care providers can diagnose TB, but have to register their patients with the National Programme before they are given the necessary anti-tuberculosis drugs.

For smear-positive TB cases KPS collects samples at the end of the intensive treatment phase (2 months for standard treatment) and 1 month before the end of treatment, to confirm smear conversion. Review samples are collected at the household if necessary. All smear-positive review sputa are cultured as are all end of treatment specimens.

### Data Collection

All consenting patients starting TB treatment were interviewed by KPS staff to record information on clinical symptoms, socio-demographic background information and history of TB. Information on previous TB treatment was recorded from the KPS database and self-reports by the patient. If a patient defaulted or died before completion of treatment, they were traced or information was collected from reliable informants. From 1988 HIV testing has been offered by trained KPS field staff at diagnosis, and from 2005 onwards self-reports of ART usage have also been recorded. All data were double entered and verified.

### Analysis

TB episodes were classified as pulmonary or extra-pulmonary. Patients with mixed pulmonary and extra-pulmonary disease were classified as pulmonary. For the main incidence analysis we included all new episodes of smear-positive pulmonary TB in adults (age 15+) from 1988 onwards, in line with our previous report from this setting, because case ascertainment and diagnosis was most constant over time for this group. [17] Adult population denominators for Karonga District were acquired from our whole population survey data for the 1980s, and from national censuses from 1998 and 2008, assuming exponential growth between each census. [21] As many TB patients are started on treatment without any laboratory confirmation but based on X-ray or clinical indication, the incidence analyses were repeated with all new patients.

The drug resistance analysis included all culture-positive TB patients (including <15 year olds) who had drug sensitivity information available since 1988. “Initial” resistance was defined as resistance to an antituberculous drug in a confirmed *M.tuberculosis* isolate from a sputum sample taken before, or within the first month, of treatment from a patient with no previous treatment for TB. A separate analysis was done for those who had previously been treated (sometimes called “acquired” resistance). [28] Multi-drug resistance (MDR) was defined as resistance to both isoniazid and rifampicin.

We defined a patient as having a “clustered” strain if the strain had an RFLP pattern identical to that seen in another patient in the previous 5 years. Other strains were classified as unique.

Logistic regression was used to investigate associations between key variables and the presence of initial resistance to INH. TB cases with confirmed MDR were investigated in detail.

## Results

### Incidence

Between January 1988 and June 2011 6172 episodes of TB were registered in adults, of which 3748 (61%) had some form of laboratory confirmation (at least 1 positive smear or culture, excluding single scanty positive smears, or positive histology), which means 39% of patients were started on empirical TB treatment based on X-ray and/or clinical symptoms alone.

**Figure 1:**
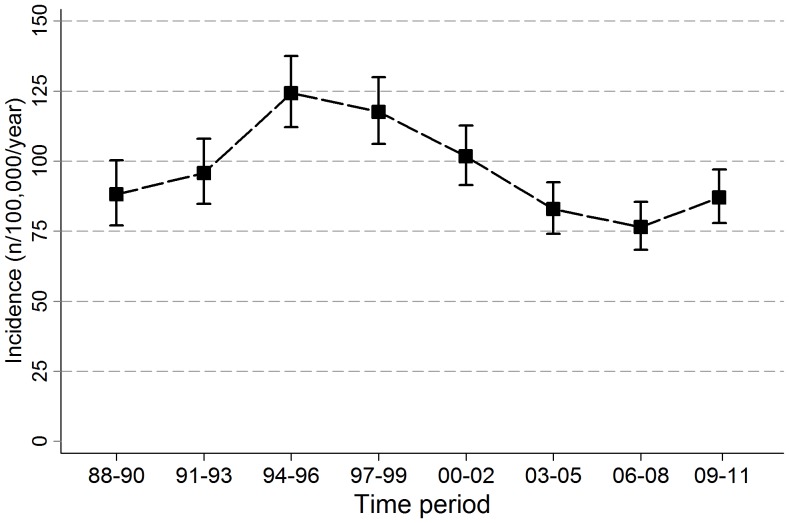
Incidence of new smear-positive pulmonary TB in Karonga District in adults. Error bars indicate 95% confidence intervals.

After excluding recurrent episodes (n = 419), episodes with extrapulmonary involvement only (n = 146) and smear-negative TB (n = 591), we included 2592 episodes of new smear-positive pulmonary TB in the analysis. Figure 1 illustrates the trends in TB incidence, with the HIV-induced peak in the mid 90′s. Since then incidence has declined steeply, and has stabilized at a level below that of 1988, the early years of the HIV-epidemic. [22].

A similar trend was seen when all new adult cases were included, a peak around 280/100,000/year in the 1990’s, but a steady decline to 136/100,000/year recently, well below the 206/100,000/year recorded in 1988–1990. The 33 cases (23 new smear-positives) that were identified during the immunological survey which started in 2008 are included in the analyses.

The proportion of all TB episodes that were due to recurrence (i.e. a new episode after treatment was completed) rose from 8% in 88–90 to 13% in 00–02, but has remained stable since.

### Drug Resistance

Drug sensitivity information was available for a total of 3132/3307 (95%) of culture-positive episodes (including 71 from <15 year olds) that occurred between January 1988 and December 2010. Of these, 2643 had information on initial resistance status (i.e. first episode of TB for that patient and drug resistance test information available from a sample collected before one month of treatment was completed).

The proportions of TB cases with initial isoniazid or multi-drug resistance are shown in table 3. Overall fewer than 6% (5.8%, 154/2643) of cases had initial isoniazid resistance and 0.6% (15/2643) had initial MDR resistance. Resistance to isoniazid was higher 15.6% (49/314) in cases that had been treated for TB before, and acquired MDR was 0.9% (3/314). There was no evidence of any increase in drug resistance over time.

**Table 3:** Anti-Tuberculosis drug resistance trends, 1988–2010.

	Initial % (n/N)		Recurrent TB	
Year	INH	(MDR) INH+RIF	INH	(MDR) INH+RIF
1988–1990	8.9 (22/246)	0.4 (1/246)	34.8 (8/23)	0 (0/23)
1991–1993	4.5 (12/267)	0 (0/267)	20.0 (5/25)	0 (0/25)
1994–1996	5.1 (14/277)	0.4 (1/277)	17.2 (5/29)	3.5 (1/29)
1997–1999	6.0 (33/555)	0.9 (5/555)	9.0 (6/67)	0 (0/67)
2000–2002	4.4 (20/457)	0.4 (2/457)	15.7 (8/51)	0 (0/51)
2003–2005	6.8 (26/382)	1.1 (4/382)	14.8 (8/54)	0 (0/54)
2006–2008	5.1 (16/317)	0.3 (1/317)	15.9 (7/44)	0 (0/44)
2009–2010	7.8 (11/142)	0.7 (1/142)	9.5 (2/21)	9.5 (2/21)^a^
Total	5.8 (154/2643)	0.6 (15/2643)	15.6 (49/314)	0.9 (3/314)

n = number resistant, N = total number tested, INH = isoniazis, RIF = Rifampcin, MDR = Multi-drug resistance.

a confidence interval for estimate = 0–23%.

Table 4 shows the association between patient characteristics and initial or acquired resistance to isoniazid. The proportions with initial INH resistance were similar in males and females, in those with different types of TB and by HIV/ART status. There was no consistent trend with age, with the lowest resistance levels in the age group 25–34. There was no evidence of an association between drug resistance and whether the RFLP-fingerprint had been previously seen in that population (i.e. was clustered) or not.

**Table 4:** Association between patients’ characteristics and resistance to INH.

	Initial resistance^a^		Recurrent TB	
Background Characteristics	% resistance (n/N)	OR (95% CI)^e^	% resistance (n/N)	OR (95% CI)^e^
Sex				
Female	5.1 (69/1350)	Ref	13.8 (21/152)	Ref
Male	6.6 (85/1293)	1.29 (0.93–1.79)	17.3 (28/162)	1.26 (0.67–2.37)
Age				
<15	10.2 (6/59)	2.39 (0.97–5.94)	20.0 (1/5)	1.21 (0.12–12.7)
15–24	5.9 (22/372)	1.37 (0.80–2.36)	9.5 (2/21)	0.56 (0.12–2.70)
25–34	4.2 (39/929)	Ref	14.8 (17/115)	Ref
35–44	7.8 (49/632)	1.89 (1.23–2.93)	16.5 (13/79)	1.04 (0.46–2.35)
≥45	5.8 (38/651)	1.38 (0.87–2.19)	17.0 (16/94)	1.14 (0.53–2.46)
TB Type				
Pulmonary^b^	5.9 (147/2509)	Ref	16.0 (49/306)	–
Extra-pulmonary only	5.2 (7/134)	0.92 (0.42–2.04)	0 (0/8)	–
Sputum Status				
smear-negative	5.9 (41/690)	Ref	8.7 (6/69)	Ref
smear-positive	5.8 (113/1953)	0.99 (0.68–1.44)	17.6 (43/245)	2.29 (0.91–5.75)
HIV-negative	5.8 (45/773)	Ref	21.7 (21/97)	Ref
HIV-positive	6.0 (62/1027)	1.09 (0.73–1.64)	10.4 (16/154)	0.54 (0.25–1.18)
HIV-pos – not on ART^c^	7.1 (7/99)	Ref	11.1 (1/9)	Ref
HIV-pos – on ART^c^	11.4 (8/70)	1.70 (0.59–4.92)	5.0 (1/20)	0.42 (0.02–7.59)
Clustered with other case in the past^d^				
Unique RFLP fingerprint	5.9 (32/544)	Ref	11.5 (6/52)	Ref
Clustered RFLP fingerprint	6.3 (62/982)	1.11 (0.70–1.75)	12.2 (16/131)	1.01 (0.36–2.83)

a ‘Initial resistance’ defined as resistance found in sample from patient with less than 1 month of TB treatment and experiencing their first TB episode.

b this includes mixed pulmonary and extra-pulmonary TB (n = 87 for initial, n = 7 for recurrences);

c only for HIV-positive episodes occurring from 2005 onwards;

d N is lower because RFLP not available for all cases;

e adjusted for period; Ref = reference category.

Details of the 18 patients with MDR are shown in table 5. MDR TB was seen at low levels throughout the study period, over a wide age range, and in HIV-positive and HIV-negative individuals. Case fatality rates were high (46.7% (7/15) with initial MDR, compared to 21.8% (720/3307) of all culture positive cases). RFLP results were available for 12 patients with MDR TB; four patients diagnosed with initial MDR TB between 1994 and 2003 shared a common strain, kps_43. This was only found in two other patients; a 1995 patient with initial resistance to INH (and streptomycin), and a patient in 1999 with a fully sensitive strain. There were no known epidemiological links between the 4 MDR patients. The two patients with MDR TB due to strain kps_12 were husband and wife. Strain kps_12 is one of the more common strains in the area [32], and initial INH resistance was found in 34% (9/41) of all episodes with this strain.

**Table 5:** Details of MDR cases.

MDR Person	Type of MDR	Year	Sex	Age	HIV/ART status	Outcome	RFLP Strain	Unique or clustered^a^	Place in cluster (total cases)	Years since strain last seen	Years until strain seen again	(M)DR in previous strains	(M)DR in later strains
1	Initial	1989	F	18	HIV−	Completed							
2	Initial	1994	M	52		Died	KPS_43	Unique	1(6)	0	0	–	Yes – MDR
3	Initial	1997	M	55	HIV−	Died	KPS_43	Clustered	3(6)	2	1	Yes – MDR	Yes – MDR
4	Initial	1997	F	18		Died							
5	Initial	1997	F	38	HIV−	Completed	KPS_43	Clustered	4(6)	1	0	Yes – MDR	Yes – MDR
6	Initial	1998	M	49	HIV−	Died	KPS_182	Unique	1(1)	0	0		
7	Initial	1998	M	29		Left	KPS_369	Unique	1(1)	0	0		
8	Initial	2001	M	34		Completed	KPS_137	Clustered	4(6)	2	1	No	No
9	Initial	2002	M	41		Died							
10	Initial	2003	F	21	HIV−	Completed	KPS_406	Clustered	5(14)	0	0	No	No
11	Initial	2003	M	57		Died	KPS_43	Clustered	6(6)	5	4	Yes – MDR	Last strain seen
12	Initial	2004	F	51	HIV+ no ART	Completed	KPS_12	Clustered	39(49)	0	0	Yes – initial INH	
13	Initial	2004	F	26		Completed	KPS_519	Unique	1(1)	0	0		
14	Initial	2008	M	59	HIV−	Unknown	KPS_12	Clustered	49(49)	0	0	Yes – MDR	Last strain seen^b^
15	Initial	2009	M	35	HIV−	Died							
													
1	Previous TB	1994	M	31		Died							
2	Previous TB	2010	F	31	HIV+ ART>6m	Completed							
3	Previous TB	2010	M	45	HIV−	Failed							

Outcomes of 18 MDR cases: Completed 39% (7/18), Died 44% (8/18), Left/Failed/Unknown 17% (3/18).

a clustered with another strain in previous 5 years;

b Last RFLP done in 2008, so cluster could have continued since.

## Discussion

Our data show that with good support, TB control in Karonga District has kept drug resistance levels low, including in recurrent cases. Furthermore, despite an increase in TB incidence following the rise in HIV prevalence, and 60% of the TB cases being HIV-positive, TB incidence levels are now lower than those seen in the early years of the HIV epidemic. [17].

Our results are in line with national reports from the region which mostly show declining trends in TB incidence in recent years. [14] Similar levels of drug resistance were seen in several cross-sectional surveys in the region (Zambia, Uganda and Botswana) in the context of generalised HIV epidemics and standard smear-microscopy based DOTS programmes. [3]–[6] As in these studies, no evidence was found of an association between drug resistance and HIV status or ART use, although the numbers on ART are small in our study.

The research activities in Karonga District may have improved TB control, but we believe this is largely because we were able to ensure basic activities were carried out correctly rather than because of any high tech or costly interventions. Our system of having staff at peripheral health clinics is a form of enhanced case finding that could be replicated by providing basic training of local staff. Other, population-based, studies at KPS yield very few TB cases, suggesting that our clinic-based case detection is at acceptable levels. The immunological study may have increased case-finding in the hospital, although the proportion of all TB cases that was found on the general ward was similar in the 1997–2007 and 2008–2009 period (12.9% versus 15.7%, p = 0.3) It is also likely that a proportion of the cases identified through this sub-study would have been found through regular passive case finding at a later date, albeit more spread out over time.

The culture confirmation for all individuals suspected of having TB, and speciation plus drug sensitivity for all culture-positive cases would have had limited impact on clinical care, due to the very low levels of drug resistance in our region and intensive microscopic and clinical surveillance strategy, and are not essential for achieving the results. As an indication, approximately 5% of smear-negative culture positive cases were commenced on TB treatment after a positive culture report was available (unpublished data), the overwhelming majority having already started TB treatment on clinical suspicion prior to receiving the culture result. Thus, this is unlikely to have had a major impact on TB incidence trends.

Our sputum collecting protocol for TB suspects (three samples over two days, ‘spot-morning-spot’ [26]) is labour-intensive and maintained for study reasons. Alternative approaches, such as taking two spot-sputa on the same day, can be effective while reducing the laboratory and health staff workload. Our strategy of following up patients at their household if they failed to return to the clinic was also likely to be an important factor in the overall success [26], [33].

Throughout, we have used treatment as determined by the NTP. In the earlier years we supported treatment, but there has been no rise in resistance or incidence following the transfer of treatment services back to the NTP. Rifampicin has been in use in the intensive phase throughout and has been part of the continuation phase since 2007, but initial MDR rates have remained low, suggesting continued absence of onward MDR transmission. The proportion of episodes with resistance was similar in clustered and unclustered strains, suggesting no overall effects on transmissibility. It will be important to continue to monitor rifampicin resistance now that it is used throughout treatment, increasing selective pressure.

These data provide a comprehensive picture of TB control in a challenging setting. The reduction in incidence occurred at a time when HIV prevalence appeared to be stable [22] and before widespread use of ART. Although it could be explained by lower case detection, there is no evidence for this. Restricting the analysis to smear positive cases, for whom procedures were similar over time, should minimize this potential bias.

In Karonga District, the decline in TB incidence seems to have halted. The reasons for this are unclear. It is not explained by the extra cases identified through screening on the general wards. To bring incidence down further, more intensive screening may be needed, in high risk populations (e.g. ART clinics) or in the community. Such studies are underway in Karonga District.

### Conclusion and Recommendations

Our results re-emphasize the need for doing the ‘simple things’ well. Improving TB control should include strengthening the existing, sputum smear based DOTS infrastructure, as well as support of ART programmes, which remain the cornerstones of cost-effective, equitable and sustainable TB control in many resource-limited settings. This applies in particular for peripheral rural areas, where drug resistance levels are probably low. For these areas, low-tech and low-cost improvements to TB care would be appropriate.

## References

[1] Corbett EL, Watt CJ, Walker N, Maher D, Williams BG, et al (2003) The growing burden of tuberculosis: global trends and interactions with the HIV epidemic. Arch Intern Med 163: 1009–1021. 12742798 10.1001/archinte.163.9.1009

[2] Abdool Karim SS, Churchyard GJ, Abdool Karim Q, Lawn SD (2009) HIV infection and tuberculosis in South Africa: an urgent need to escalate the public health response. Lancet 374: 921–933. 19709731 10.1016/S0140-6736(09)60916-8 PMC2803032

[3] Lukoye D, Cobelens FG, Ezati N, Kirimunda S, Adatu FE, et al (2011) Rates of anti-tuberculosis drug resistance in Kampala-Uganda are low and not associated with HIV infection. PLoS One 6: e16130. 21249225 10.1371/journal.pone.0016130 PMC3018425

[4] Mulenga C, Chonde A, Bwalya IC, Kapata N, Kakungu-Simpungwe M, et al (2010) Low Occurrence of Tuberculosis Drug Resistance among Pulmonary Tuberculosis Patients from an Urban Setting, with a Long-Running DOTS Program in Zambia. Tuberc Res Treat 2010: 938178. 22567261 10.1155/2010/938178 PMC3335559

[5] Kenyon TA, Mwasekaga MJ, Huebner R, Rumisha D, Binkin N, et al (1999) Low levels of drug resistance amidst rapidly increasing tuberculosis and human immunodeficiency virus co-epidemics in Botswana. Int J Tuberc Lung Dis 3: 4–11. 10094163

[6] Talbot EA, Kenyon TA, Mwasekaga MJ, Moeti TL, Mallon V, et al (2003) Control of anti-tuberculosis drug resistance in Botswana. Int J Tuberc Lung Dis 7: 72–77. 12701838

[7] Wright A, Zignol M, Van Deun A, Falzon D, Gerdes SR, et al (2009) Epidemiology of antituberculosis drug resistance 2002–07: an updated analysis of the Global Project on Anti-Tuberculosis Drug Resistance Surveillance. Lancet 373: 1861–1873. 19375159 10.1016/S0140-6736(09)60331-7

[8] Evans CA (2011) GeneXpert–a game-changer for tuberculosis control? PLoS Med 8: e1001064. 21814497 10.1371/journal.pmed.1001064 PMC3144196

[9] Lawn SD, Kerkhoff AD, Wood R (2012) Location of Xpert(R) MTB/RIF in centralised laboratories in South Africa undermines potential impact. Int J Tuberc Lung Dis 16: 701. 22507934 10.5588/ijtld.12.0131

[10] Peters D, Theron G, Peter J, Dheda K (2012) Should Xpert(R) MTB/RIF be rolled out in low-income countries? Int J Tuberc Lung Dis 16: 702–703. 10.5588/ijtld.12.0034 22507936

[11] Trebucq A, Harries AD (2012) In reply to ‘Location of Xpert(R) MTB/RIF in centralised laboratories in South Africa undermines potential impact’. Int J Tuberc Lung Dis 16: 702. 10.5588/ijtld.12.0131-2 29070250

[12] Trebucq A, Harries AD, Rieder HL (2012) In reply to ‘Should Xpert(R) MTB/RIF be rolled out in low-income countries?’. Int J Tuberc Lung Dis 16: 703–704. 29070251 10.5588/ijtld.12.0034-2

[13] Trebucq A, Enarson DA, Chiang CY, Van Deun A, Harries AD, et al (2011) Xpert(R) MTB/RIF for national tuberculosis programmes in low-income countries: when, where and how? Int J Tuberc Lung Dis 15: 1567–1572. 22005110 10.5588/ijtld.11.0392

[14] WHO (2010) Global tuberculosis control: WHO report 2010. Geneva: World Health Organisation.

[15] Crampin AC, Glynn JR, Fine PE (2009) What has Karonga taught us? Tuberculosis studied over three decades. International Journal of Tuberculosis & Lung Disease 13: 153–164. 19146741 PMC3272402

[16] Floyd S, Molesworth A, Dube A, Banda E, Jahn A, et al (2010) Population-level reduction in adult mortality after extension of free anti-retroviral therapy provision into rural areas in northern Malawi. PLoS One 5: e13499. 20976068 10.1371/journal.pone.0013499 PMC2957442

[17] Glynn JR, Crampin AC, Ngwira BM, Mwaungulu FD, Mwafulirwa DT, et al (2004) Trends in tuberculosis and the influence of HIV infection in northern Malawi, 1988–2001. Aids 18: 1459–1463. 15199323 10.1097/01.aids.0000131336.15301.06

[18] Glynn JR, Jenkins PA, Fine PE, Ponnighaus JM, Sterne JA, et al (1995) Patterns of initial and acquired antituberculosis drug resistance in Karonga District, Malawi. Lancet 345: 907–910. 7707817 10.1016/s0140-6736(95)90016-0

[19] Warndorff DK, Yates M, Ngwira B, Chagaluka S, Jenkins PA, et al (2000) Trends in antituberculosis drug resistance in Karonga District, Malawi, 1986–1998. Int J Tuberc Lung Dis 4: 752–757. 10949327

[20] Mwaungulu FD, Crampin AC, Kanyongoloka H, Mwafulirwa DT, Mwaungulu JN, et al (2002) Antituberculosis drug resistance in Karonga District: pattern and trend, 1986–2001. Malawi Medical Journal 13: 3–6.

[21] Malawi Population and Housing Census. Zomba: National Statistics Office, Malawi.

[22] White RG, Vynnycky E, Glynn JR, Crampin AC, Jahn A, et al (2007) HIV epidemic trend and antiretroviral treatment need in Karonga District, Malawi. Epidemiol Infect 135: 922–932. 17217548 10.1017/S0950268806007680 PMC2870652

[23] Jahn A, Floyd S, Crampin AC, Mwaungulu F, Mvula H, et al (2008) Population-level effect of HIV an adult mortality and early evidence of reversal after introduction of antiretroviral therapy in Malawi. Lancet 371: 1603–1611. 18468544 10.1016/S0140-6736(08)60693-5 PMC2387197

[24] Ponnighaus JM, Fine PE (1986) The Karonga prevention trial–which BCG? Lepr Rev 57 Suppl 2285–292. 3553801 10.5935/0305-7518.19860082

[25] Ponnighaus JM, Fine PE, Bliss L, Gruer PJ, Kapira-Mwamondwe B, et al (1993) The Karonga Prevention Trial: a leprosy and tuberculosis vaccine trial in northern Malawi. I. Methods of the vaccination phase. Lepr Rev 64: 338–356. 8127221 10.5935/0305-7518.19930039

[26] Cuevas LE, Yassin MA, Al-Sonboli N, Lawson L, Arbide I, et al (2011) A multi-country non-inferiority cluster randomized trial of frontloaded smear microscopy for the diagnosis of pulmonary tuberculosis. PLoS Med 8: e1000443. 21765808 10.1371/journal.pmed.1000443 PMC3134460

[27] Ngwira B, Chagaluka S, Warndorff D, Branson K, Lucas S, et al (2002) Development of a scoring system for the diagnosis of tuberculous lynphadenitis. Malawi Med J 13: 14–16.

[28] WHO (2009) Guidelines for surveillance of drug resistance in tuberculosis, 4th Edition. Geneva: World Health Organisation.

[29] van Embden JD, Cave MD, Crawford JT, Dale JW, Eisenach KD, et al (1993) Strain identification of Mycobacterium tuberculosis by DNA fingerprinting: recommendations for a standardized methodology. J Clin Microbiol 31: 406–409. 8381814 10.1128/jcm.31.2.406-409.1993 PMC262774

[30] Glynn JR, Crampin AC, Yates MD, Traore H, Mwaungulu FD, et al (2005) The importance of recent infection with Mycobacterium tuberculosis in an area with high HIV prevalence: a long-term molecular epidemiological study in Northern Malawi. J Infect Dis 192: 480–487. 15995962 10.1086/431517

[31] NTP (2007) Malawi National Tuberculosis Control Programme Manual 6th Edition.

[32] Glynn JR, Crampin AC, Traore H, Chaguluka S, Mwafulirwa DT, et al (2008) Determinants of cluster size in large, population-based molecular epidemiology study of tuberculosis, northern Malawi. Emerg Infect Dis 14: 1060–1066. 18598626 10.3201/eid1407.060468 PMC2600342

[33] Crampin AC, Floyd S, Mwaungulu F, Black G, Ndhlovu R, et al (2001) Comparison of two versus three smears in identifying culture-positive tuberculosis patients in a rural African setting with high HIV prevalence. Int J Tuberc Lung Dis 5: 994–999. 11716350

